# Fish-derived biomaterials for tissue engineering: advances in scaffold fabrication and applications in regenerative medicine and cancer therapy

**DOI:** 10.7150/thno.109186

**Published:** 2025-04-21

**Authors:** Seoyul Jo, Hanjun Hwangbo, Nacionales Francis, JaeYoon Lee, Mohan Pei, GeunHyung Kim

**Affiliations:** 1 Department of Precision Medicine, Sungkyunkwan University School of Medicine (SKKU-SOM), Suwon 16419, Republic of Korea.; 2 Institute of Quantum Biophysics, Department of Biophysics, Sungkyunkwan University, Suwon, Gyeonggi-do 16419, Republic of Korea.; 3 Biomedical Institute for Convergence at SKKU (BICS), Sungkyunkwan University, Suwon 16419, Republic of Korea.

**Keywords:** fish-derived biomaterials, tissue engineering, scaffold structure, stem cell activation, cancer therapeutics

## Abstract

Fish-derived biomaterials, such as collagen, polyunsaturated fatty acids, and antimicrobial peptides, have emerged as promising candidates for scaffold development in stem cell therapies and tissue engineering due to their excellent biocompatibility and low immunogenicity. Although good bioactivity is a prerequisite for biomedical substitutes, scaffold design is necessary for the successful development of bioconstructs used in tissue regeneration. However, the limited processability of fish biomaterials poses a substantial challenge to the development of diverse scaffold structures. In this review, unlike previous reviews that primarily focused on the bioactivities of fish-derived components, we placed greater emphasis on scaffold fabrication and its applications in tissue regeneration. Specifically, we examined various cross-linking strategies to enhance the structural integrity of fish biomaterials and address challenges, such as poor processability, low mechanical strength, and rapid degradation. Furthermore, we demonstrated the potential of fish scaffolds in stem cell therapies, particularly their capacity to support stem cell growth and modulate the cellular microenvironment. Finally, this review provides future directions for the application of these scaffolds in cancer therapy.

## Introduction

Tissue engineering (TE) and regenerative medicine are transformative fields that integrate materials engineering, biology, and clinical innovation to repair or replace damaged tissues and organs. Central to these fields are bioactive materials and biomedical scaffolds, which replicate the extracellular matrix (ECM) by providing structural support and essential biochemical and physical cues for cell activation, adhesion, proliferation, and differentiation 1, 2.

Among bioactive materials, fish-derived biomaterials have gained prominence as sustainable and biocompatible resources for scaffold fabrication, offering both therapeutic benefits, and addressing environmental and ethical concerns 3, 4. In particular, biomaterials, including collagen, antimicrobial peptides (AMPs), hydroxyapatite, and omega-3 fatty acids demonstrate exceptional bioactivity. Fish-derived collagen offers a safe and ethical alternative to mammalian collagen, whereas hydroxyapatite exhibits osteoconductive properties that are critical for bone and muscle regeneration 5-9. Bioactive components, such as omega-3 fatty acids and AMPs, further enhance the functionality of scaffolds by promoting anti-inflammatory effects, preventing infections, and fostering a regenerative microenvironment conducive to stem cell activation and tissue repair 10-13.

Scaffold design using fish-derived biomaterials substantially influences tissue regeneration outcomes. Nanofiber scaffolds mimic the ECM, promoting cell adhesion and nutrient diffusion, whereas 3D-printed scaffolds allow precise control over geometry and pore size, optimizing mechanical properties and mass transport 2. Nanofibrous composite scaffolds integrating fish-derived collagen with other biomaterials have demonstrated enhanced bioactivity, facilitating cellular processes such as adhesion, proliferation, and differentiation across various tissue types 14.

Moreover, tailoring important properties, such as porosity, surface roughness, and mechanical characteristics, can enable the development of scaffolds for specific tissue types, thereby amplifying the regenerative potential of fish-derived biomaterials. Lyophilized scaffolds are characterized by a highly porous architecture that supports cellular migration and angiogenesis, making them suitable for applications requiring enhanced tissue regeneration. These scaffolds have demonstrated potential in promoting targeted differentiation and facilitating the repair of complex tissue interfaces such as osteochondral defects 15.

Notably, the integration of fish-derived biomaterials with advanced scaffold designs has proven effective in enhancing biofunctional properties, particularly by addressing limitations such as insufficient mechanical strength and rapid degradation 16. Despite these advancements, achieving complex 3D structures remains challenging due to difficulties in material stability and the precise control needed for geometry and pore size. Strategies, such as crosslinking or blending with synthetic materials, can help overcome these challenges, enabling the fabrication of robust scaffolds with improved structural and functional integrity, thus enhancing their suitability for TE applications 17, 18. Approaches like methacrylation to create photocrosslinkable bioinks 19-21 or dual-crosslinking methods combining covalent and ionic interactions 22 have demonstrated the potential to enhance mechanical properties and stability, facilitating the construction of complex, bioactive scaffolds suitable for diverse regenerative applications.

Furthermore, functionalizing scaffolds with bioactive molecules, such as growth factors and ECM-derived peptides, mimics the dynamic native tissue environment and advances their regenerative capacity. The bioactivities of fish-derived biomaterials, provide substantial advantages in TE by fostering a favorable microenvironment for tissue regeneration. When integrated with scaffold biofabrication strategies, such as electrospinning 23, 3D printing 24, or lyophilization 25, and combined with crosslinking techniques 26, these biomaterials can be precisely tailored to support specific cellular activities. The anti-inflammatory properties of fish-derived biomaterials help to modulate immune responses, whereas their antimicrobial effects prevent infections, thereby enhancing the regenerative environment for stem cell differentiation. In addition, the angiogenic potential of fish-based biomaterials stimulates blood vessel formation, promoting tissue vascularization 27. The synergistic combination of these bioactive properties, biofabrication methods, and mechanical enhancements facilitates the activation of stem cells, promoting tissue regeneration and functional recovery for various therapeutic applications.

**Figure 1:**
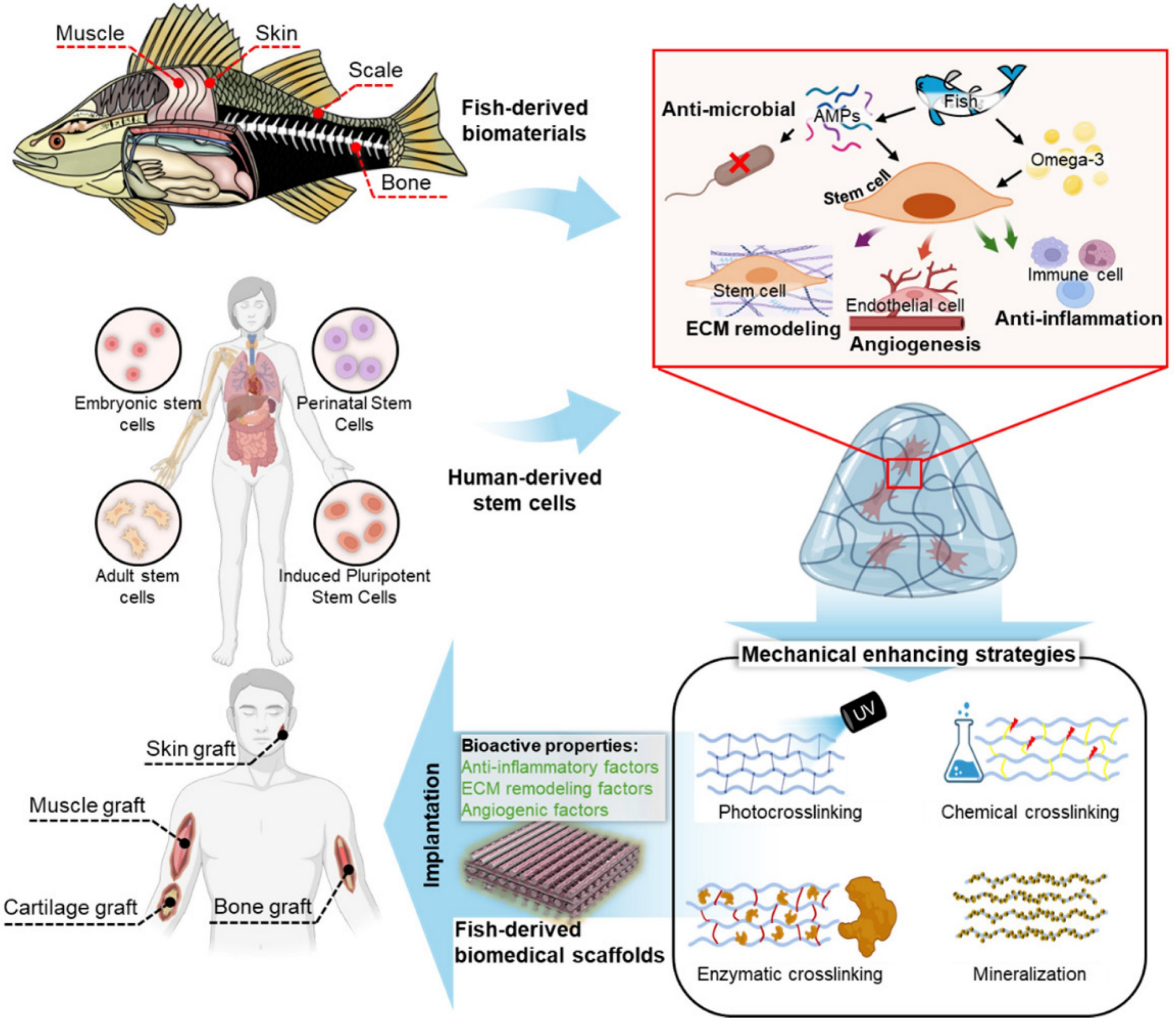
Fish-derived biomaterials for scaffold fabrication in stem cell therapies: enhancing biocompatibility, supporting cell growth, and promoting tissue regeneration. ECM, extracellular matrix.

This review comprehensively examined the applications of fish-derived biomaterials in scaffold design for stem cell activation and therapeutics, emphasizing their role in promoting critical biological processes, such as angiogenesis, anti-inflammatory responses, and ECM remodeling (Figure 1). Additionally, this review explores innovative strategies to address these limitations and highlights the potential of fish-derived scaffolds in emerging fields, such as cancer therapeutics.

## Composition and biological potentials of fish-based biomaterials

Bioactive compounds, such as omega-3 fatty acids and proteins, that constitute fish-derived biomaterials exhibit favorable biocompatibility, low immunogenicity, and support cellular activities, such as proliferation, differentiation, and tissue repair. These biomaterials, obtained from various tissues such as the skin, scales, bones, and muscles, offer promising avenues in TE, wound healing, and regenerative medicine because of their potential to modulate inflammatory responses, promote angiogenesis, and enhance ECM remodeling. In the following sections, the composition and biological properties of biomaterials derived from different fish tissues are discussed.

### Fish-derived biomaterials

#### Skin

Similar to mammalian skin, fish skin is primarily composed of type I collagen. However, fish skin collagen differs from mammalian collagen in terms of amino acid composition. Specifically, FC has a lower molecular weight and lower levels of hydroxyproline and proline, which contribute to the stability of the triple-helical structure of collagen 28. Owing to the lower amounts of these components in FC, they tend to have reduced structural stability compared with that of mammalian collagen. Furthermore, differences in the amino acid composition affect the denaturation temperature of collagen. Owing to lower levels of hydroxyproline and proline, the triple-helical structure of collagen unravels easily at high temperatures, resulting in a lower denaturation temperature for FC than mammalian collagen 29. This adaptation allows fish to function efficiently in cold environments.

Unlike the stratum corneum, which is the outermost layer of human skin, the skin of fish is covered by a mucus layer. The mucus layer serves as the first line of defense against pathogens and is involved in regulating various physiological processes, such as resistance to pathogens, ion and osmotic balance, movement, and reproduction 30. Additionally, fish skin contains several bioactive peptides. Bioactive peptides are composed of 3-30 amino acids and participate in various biological functions, including anti-cancer, antioxidant, anti-inflammatory, immune regulatory, anti-atherosclerotic, antihypertensive, and antimicrobial activities 31. This unique mechanism of fish may be an evolutionary characteristic to cope with aquatic pathogens in their environment.

An important component of fish skin is its lipid content, particularly polyunsaturated fatty acids (PUFAs). PUFAs are fatty acids that contain two or more double bonds, with omega-6 and omega-3 fatty acids being prominent examples and omega-3 fatty acids being the most abundant in fish. Omega-3 fatty acids are primarily categorized into three forms: alpha-linolenic (ALA), eicosapentaenoic (EPA), and docosahexaenoic (DHA) acid, which are abundant in fish-derived biomaterials 32.

#### Scale

Fish scales (FSs) are complex structures that play vital roles in aquatic organisms. FSs consist of a combination of organic and inorganic materials. The organic part, making up about 41-45% of the scale, is mainly type I collagen, along with small amounts of other proteins, lipids, and carbohydrates. 33. This organic matrix provides flexibility to scale structures. The inorganic component, approximately 38-46% of the scale, consists mainly of hydroxyapatite, a calcium-deficient form of the mineral that imparts rigidity and strength to scales 34.

In particular, collagen from FS is considered a valuable biomaterial source because of its orderly orientation and the absence of zoonotic infections and religion-related complications 35. Gelatin is obtained by breaking the hydrogen bonds in collagen polypeptide chains through thermal hydrolysis. This process enhances joint and bone health, increases bone marrow density, and offers an alternative biomaterial for patients with skeletal conditions like osteoporosis. Additionally, hydroxyapatite has a similar function; in vitro studies have shown that it promotes cellular calcium uptake, suggesting its role in bone metabolism 36. Finally, chitin, a polysaccharide with antibacterial and antifungal properties, can be attained from FSs. Depending on the species, chitin yield from fish could reach up to 45% 37.

#### Bone

Fish bones, which provide structural support and enable mobility, offer potential as biomaterials for scaffold fabrication in tissue regeneration, composed of both organic and inorganic components. The organic matrix includes type I collagen, other proteins, and trace amounts of lipids and carbohydrates, whereas the inorganic portion primarily consists of hydroxyapatite (Ca₁₀(PO₄)₆(OH)₂), a key mineral found in human bone. This composition contributes to the mechanical properties and bioactive potential, with water, organic materials, and minerals comprising 10-55%, 35-70%, and 30-65% of their total weight, respectively 38. Beyond the mechanical functions, fish bones act as reservoirs for essential minerals like calcium and phosphate, playing a critical role in metabolic regulation and contributing to blood cell production through their bone marrow.

These attributes position fish bones as a promising source of biomaterials for TE applications 39. Their composition, featuring type I collagen and hydroxyapatite, closely resembles that of native human bone, offering intrinsic biocompatibility and osteoconductivity. Hydroxyapatite derived from fish bones can be processed into scaffolds that support bone cell adhesion and proliferation, while collagen components enhance scaffold flexibility and promote tissue formation 40. Moreover, the natural hierarchical porosity of fish bones can be preserved or adjusted during fabrication, enabling the creation of scaffolds with interconnected pores that facilitate critical processes such as cell infiltration, nutrient diffusion, and waste removal 41. Advances in processing technologies, including decellularization, demineralization, and biofabrication strategies, have further expanded the utility of fish bones as bioactive scaffolds.

#### Muscle

Fish muscle is a highly specialized tissue that plays a crucial role in aquatic locomotion. Comprising 15-25% of the total protein in fish consists of myofibrillar (50-60%), sarcoplasmic (30%), and stromal (10-20%) proteins 42. Fish muscle fibers are generally shorter and contain less connective tissue than those of terrestrial animals, contributing to the soft texture of fish flesh, which offers a new context for use in biomedical applications. These fibers are organized into segments called myotomes, which are separated by thin sheets of connective tissue known as myocommata 43.

A vital consideration in utilizing fish muscle protein for various biomedical, pharmaceutical, and fabrication applications is its rich amino acid content, most specifically, lysine (9.3%), leucine (8.9%), and isoleucine (6.4%), which can potentially regulate age-induced bone loss 44. Protein content varies within species, with cod and salmon muscles being composed of 18.2% and 23.5% of protein, respectively 45. However, moisture content, an important factor in protein extraction, can be as high as 84.6% in flounder 45. The functional properties of these proteins, including their antioxidant and bioactive potential, still make them valuable for the development of nutraceuticals and therapeutic compounds, especially through methods such as enzymatic hydrolysis for bioactive peptide extraction.

### Biological properties of fish-derived biomaterials for stem cell activation

**Figure 2:**
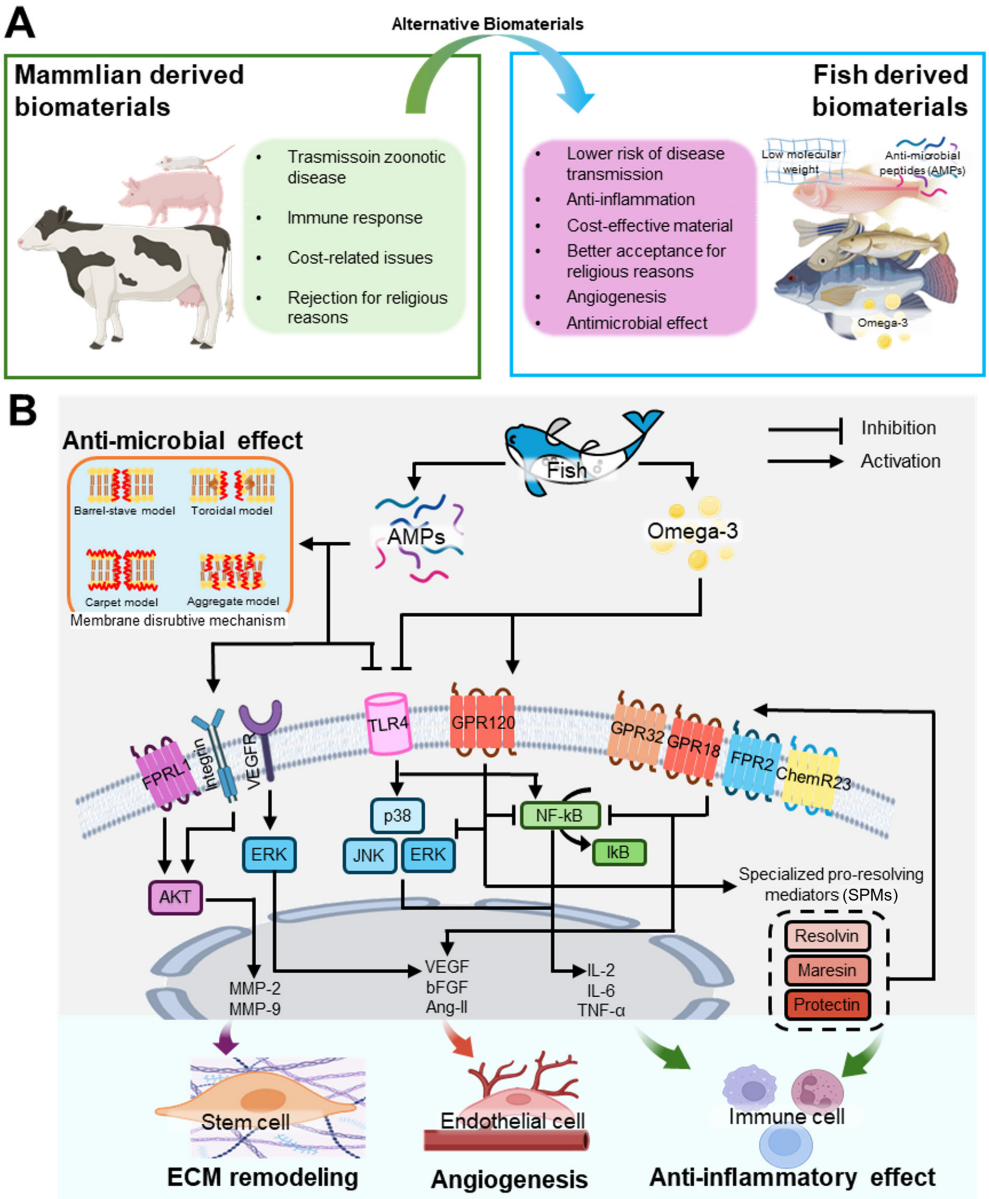
** Bioactivities of stem cells cultured on fish-derived biomaterials.** (**A**) Comparative analysis of mammalian and fish-derived biomaterials, and (**B**) intercellular pathways activated by fish-derived biomaterials that stimulate cellular responses, such as antimicrobial activity, extracellular matrix (ECM) remodeling, angiogenesis, and anti-inflammatory effects.

Mammalian-derived biomaterials are widely used in scaffold fabrication for tissue regeneration owing to their high biocompatibility and structural similarity to human tissues. However, these materials have several limitations, including disease susceptibility spread among terrestrial animals, high cost, immune responses, and religious restrictions (Figure 2A). In contrast, fish-derived biomaterials are more cost-effective, show a lower risk of disease transmission, and are more accepted for religious reasons, making them promising alternatives to mammalian biomaterials (Figure 2A). Additionally, fish-derived materials have a low molecular weight, offering enhanced bioavailability compared with that of mammalian sources 46. These bioactive materials including omega-3 fatty acids and AMPs can activate stem cells and induce various intercellular activities shown in Figure 2B.

#### Anti-inflammatory capacities

Omega-3 fatty acids in fish, such as DHA and EPA, play crucial roles in reducing inflammation. These fatty acids inhibit toll-like receptor 4 (TLR4) and enter the cell through GPR120 47-49. Through this process, they can suppress nuclear factor kappa B (NF-κB) and mitogen-activated protein kinase (MAPK) signaling pathways, both of which are involved in producing inflammatory cytokines, such as tumor necrosis factor (TNF)-α and interleukin (IL)-6, thus mitigating inflammatory responses 49-54.

NF-κB is a key transcription factor that regulates inflammation and immune responses within cells. NF-κB is primarily activated through the degradation of IκBα triggered by its site-specific phosphorylation by a multi-subunit IκB kinase (IKK) complex 50, 55. Activated NF-κB promotes the production of inflammatory cytokines, such as IL-1, IL-6, IL-12, TNF-α, and chemokines, facilitating the activation of various immune cells 50, 51. Additionally, cytokines, such as IL-1β and TNF-α, act through Toll-like, IL-1 (TIR), or TNF receptors. Furthermore, the MAPK signaling pathway is involved in the activation of the receptors related to the inflammatory response 49, 52. The MAPK pathway is primarily composed of extracellular signal-regulated kinases (ERK), c-Jun N-terminal kinases (JNK), and p38, which are three key signaling proteins. Among these, JNK and p38 MAPK proteins promote the expression of proinflammatory cytokines and apoptosis, thereby inducing an inflammatory response 49, 53, 54.

Omega-3 fatty acids inhibit the degradation of IκBα, thus blocking the activation of NF-κB, and prevent the phosphorylation of signaling proteins involved in the MAPK pathway, thereby suppressing excessive activation of the MAPK pathway 48, 56-58. Furthermore, DHA and EPA can be converted into specialized pro-resolving mediators (SPMs). SPMs are bioactive substances derived from essential fatty acids that regulate inflammation and promote resolution, aiding in the removal of pathogens, dead cells, and debris from inflamed tissues 59. Major constituents of SPMs include resolvins, protectins, and maresins derived from DHA and EPA 60, 61.

Fish AMPs can also induce anti-inflammatory effects 62-64. AMPs inhibit TLR4, and prevent excessive inflammation by regulating immune cell differentiation and suppressing inflammatory responses 65, 66. Notable anti-inflammatory peptides include LL-37 and β-defensin, which exhibit anti-inflammatory effects and function as AMPs 67-70.

#### Antimicrobial capacities

Fish, especially their skin, synthesize and release AMPs in response to pathogen invasion 71, 72. These AMPs destroy pathogens by direct killing, membrane disruption, opsonization, or inhibition of DNA/RNA/protein synthesis. AMPs, including pleurocidin, β-defensin, hepcidin, cathelicidins, and piscidin, are particularly abundant in the mucous layer of fish skin 73. These peptides are effective against gram-positive and gram-negative bacteria, fungi, and viruses 74.

AMPs damage bacterial cell membranes, allowing them to penetrate and disrupt physiological functions, ultimately leading to cell death. AMPs can penetrate the cell membrane because they typically possess a positive charge, whereas bacterial cell membranes carry a negative charge, enabling the strong adhesion of AMPs to the membrane 75-77. Once attached to the membrane, AMPs induce structural changes that form various types of membrane pores, facilitating their entry into the cells. Inside the cell, AMPs inhibit the synthesis of DNA, RNA, and proteins, and impede protein folding, enzyme activity, and cell wall synthesis, while promoting the release of lytic enzymes that disrupt cellular structures 78, 79.

Additionally, omega-3 fatty acids contribute to antimicrobial capacity. SPMs derived from DHA and EPA enhance macrophage phagocytic activity and promote the destruction of bacterial cell membranes, thereby providing an additional defense mechanism against microbial infections 80, 81.

#### Angiogenesis capacities

The omega-3 fatty acids play a crucial role in promoting angiogenesis and the formation of new blood vessels. DHA and EPA enhance the growth and proliferation of vascular endothelial cells by increasing the expression of key angiogenic regulators, such as vascular endothelial growth factor (VEGF), basic fibroblast growth factor (bFGF), and angiopoietin2 (Ang-II) 82-85. This leads to migration, differentiation, and proliferation of endothelial cells, thereby facilitating the formation of new blood vessels.

Although the mechanisms by which omega-3 fatty acids influence the expression of vascular growth factors have not been clearly elucidated, several studies have shown that omega-3 fatty acids promote vascular formation. Wang *et al.* demonstrated the vascularization capacity of omega-3 fatty acids using transgenic mice that overproduced omega-3 fatty acids 83. The researchers induced transient focal cerebral ischemia in transgenic mice and discovered that omega-3 promotes the upregulation of Ang-II in astrocytes and the release of this angiogenic regulator into the extracellular space. Additionally, Mathew and Bhonde confirmed that the expression of bFGF and VEGF is enhanced when placenta-derived mesenchymal stromal cells are cultured in a medium supplemented with DHA and EPA 82. They also demonstrated the effectiveness of omega-3 fatty acids in promoting angiogenesis by treating chick yolk sac membranes with conditioned media collected during this process.

Moreover, as previously mentioned, DHA and EPA are converted into SPMs, which not only resolve excessive inflammation but also promote tissue repair. By reducing inflammation, SPMs create a more favorable environment for endothelial cells to function effectively, thereby supporting angiogenesis and contributing to the maintenance of vascular health 86.

AMPs can also bind to various receptors to facilitate activation of the AKT pathway 87. AKT signaling is a central pathway that regulates metabolism, proliferation, cell survival, and angiogenesis in response to various extracellular cues. The activation of the AKT pathway leads to the upregulation of matrix metalloproteinases (MMPs) associated with ECM remodeling and angiogenic factors, thereby promoting the regeneration of damaged tissues and vessel formation 87, 88.

## Biomedical scaffold based on fish-derived biomaterial

### Structural effects of biomedical scaffolds on stem cell activation

Biomedical scaffolds used in stem cell therapy have been utilized as platforms for cell adhesion, proliferation, and differentiation, improving stem cell viability and function 89-91. A key advantage of these structures is their potential for customization, as various biomaterials, such as collagen, peptides, and ECMs can be used in conjunction with biofabrication processes (e.g. extrusion bioprinting, electrospinning, and stereolithography) to create a wide array of structural types that could potentially improve treatment efficiency (Table 1).

These fabrication methods offer distinct benefits for different tissue engineering applications. Extrusion bioprinting involves layer-by-layer deposition of bioinks, enabling the fabrication of scaffolds with controlled geometries and adjustable pore sizes, which are crucial for cellular migration and nutrient exchange. Electrospinning uses an electric field to draw polymer solutions into fine fibers, resulting in nanofibrous scaffolds with a high surface area and small pores that mimic the extracellular matrix, enhancing cell adhesion and directional growth 92. Stereolithography (SLA) employs a laser to crosslink photopolymerizable resins layer by layer, producing scaffolds with high resolution, intricate geometries, and uniform pore distribution, ideal for tissues requiring specific mechanical properties 93. Lyophilization freezes a biomaterial solution and removes water through vacuum drying, creating scaffolds with interconnected pores and high porosity, which support cell infiltration and tissue formation 94. Each of these methods enables precise control over scaffold morphology, allowing for the design of scaffolds suited for various regenerative medicine applications. Each of these fabrication methods enables precise control over scaffold morphology. By carefully controlling the printing parameters, these techniques enable the formation of a variety of scaffold architectures, including aligned, curved, hierarchical, and porous structures. These topological features are crucial for influencing cell behavior, guiding migration, and promoting differentiation, thereby optimizing tissue regeneration. The following section discusses how these specific architectural cues enhance scaffold functionality and support tissue engineering applications.

#### Aligned structures

**Figure 3:**
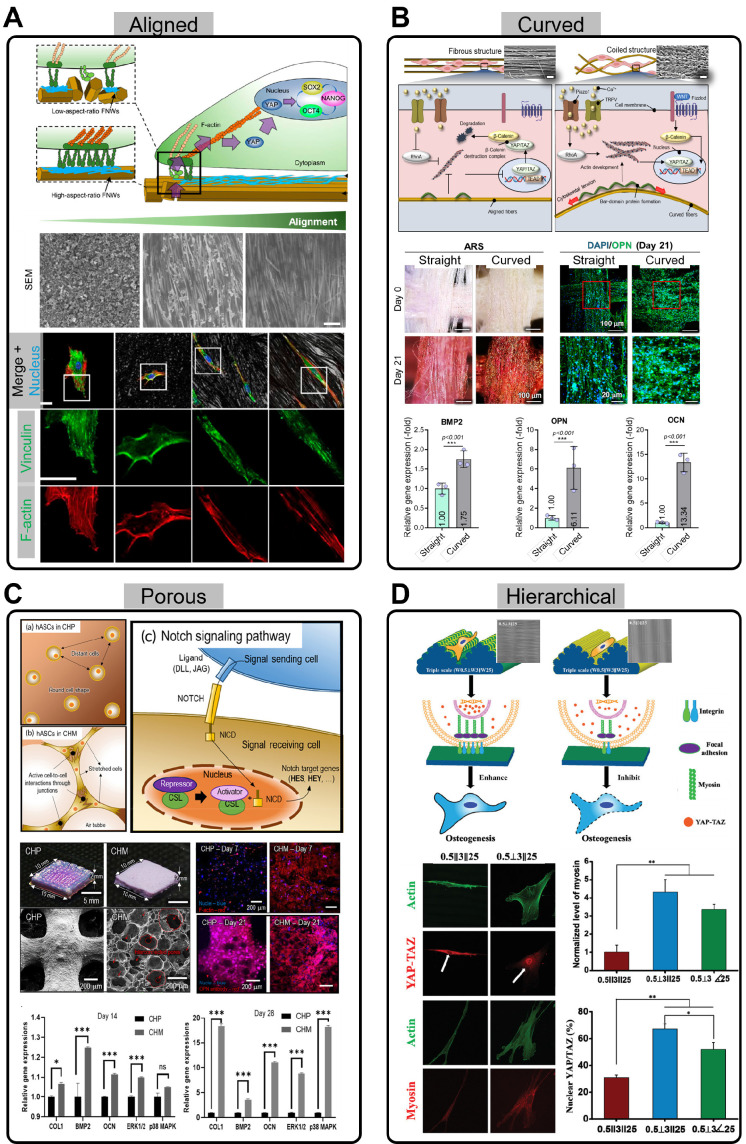
** Stem cell interactions with various scaffold structures.** (**A**) Schematic illustration showing how aligned substrates regulate the self-renewal process of human mesenchymal stem cells (hMSCs) (Adapted with permission from 95, copyright 2020). SEM images and immunofluorescence stainings of Vinculin and F-actin for various nanopatterned scaffolds. (**B**) Schematic of mechanotransduction pathways in straight and curved fibers (Adapted with permission from 102, copyright 2024). Images of Alizarin Red S (ARS) staining and DAPI/OPN, and relative expression of BMP2, OPN, and OCN in hASCs cultured on straight and curved fibers. (**C**) Illustration of the notch signaling pathway in highly porous scaffolds by stretching and active cell-to-cell interactions (Adapted with permission from 103, copyright 2022). Optical, SEM, DAPI/F-actin, and DAPI/OPN staining images of conventional scaffolds and collagen foam scaffolds, along with the relative gene expression of osteogenic genes and MAPK signaling genes (*COL1, BMP2, OCN, ERK1/2*, and *p38 MAPK*) at days 14 and 28. (**D**) Schematic showing of the interaction between hMSCs and different types of hierarchical structure (Adapted with permission from 106, copyright 2020). Immunofluorescence staining images of Actin, YAP-TAZ, and Myosin, along with fluorescence intensity of myosin and the number of cells showing nuclear localization of YAP-TAZ.

Alignment is one of the structures that play a crucial role in stem cell differentiation, proliferation, and migration. According to Song *et al.*, aligned structures have a substantial effect on stem cells, particularly in relation to the Yes-associated protein (YAP) pathway (Figure 3A) 95. Aligned structures limit the contact area between the stem cells and the substrate, preventing excessive cell spreading. This results in focal adhesions oriented along the length of the cells, which reduces cytoskeletal tension and helps maintain stem cell multipotency. These changes facilitate the translocation of the transcriptional coactivator, YAP, from the cytoplasm to the nucleus. YAP is regulated by cell-ECM interactions, and upon translocation to the nucleus, promotes the expression of genes crucial for maintaining stem cell multipotency. In particular, nuclear YAP localization enhances the expression of core stemness regulators, such as octamer-binding transcription factor 4 (OCT4), SRY-box containing gene 2 (SOX2), and Nanog homeobox (NANOG), reinforcing the self-renewal capacity of stem cells 95. Aligned structures play an important role in modulating stem cell cytoskeletal tension and facilitating nuclear translocation of YAP, ultimately promoting the retention of stem cell multipotency. This study suggests that aligned structures provide crucial mechanical signals that help maintain the physiological properties of stem cells, thereby enhancing their multipotency and self-renewal potential.

Through this mechanism, scaffolds can prevent uncontrollable differentiation of stem cells and induce them to differentiate into specific tissues. In particular, various studies have shown that aligned scaffolds are effective in promoting muscle differentiation and regeneration of stem cells 90, 96-98. In addition to muscle regeneration, aligned scaffolds that mimic the orientation of peripheral nerves enhance neural differentiation of human adipose stem cells (hASCs) 99. According to Yao *et al.*, compared to randomly distributed PCL microfibers, these aligned structures activate the phosphorylated focal adhesion kinase (p-FAK) pathway through specialized transmembrane integrin adhesions 99. This results in enhances neurotrophic effects in the neural microenvironment, providing a promising direction for improving stem cell therapy for neural applications.

#### Curved structures

Surface curvature plays a critical role in influencing tissue growth and cellular behavior 100-102. Specifically, curved regions enhance tissue growth, whereas convex areas tend to limit it 100. The interplay between the curvature and cellular dynamics is particularly notable, with cells preferentially migrating away from concave surfaces and exhibiting increased attachment, proliferation, and spreading on convex structures 100. Moreover, curvature influences ECM distribution and nuclear mechanotransduction. Mechanical forces exerted on the nucleus in response to surface curvature increase Lamin A levels, which are associated with the promotion of osteogenic differentiation. Such nuclear responses reflect how the curvature regulates intracellular signaling pathways and stem cell fate. The favorable effects of curved substrates include enhanced stem cell activity, such as improved proliferation, differentiation, and alignment. These microcurvature-induced changes in stem cell behavior highlight the importance of geometrical cues in the designing biomaterials for tissue-engineering applications.

Recently, Pei *et al.* introduced a stable die-swell extrudate biofabrication technique to produce micro-sized PCL fibers with a coiled morphology (Figure 3B) 102. When hASCs were cultured on this coiled substrate, they exhibited substantial upregulation of the Hippo and Wnt/β-catenin signaling pathways, and stretch-activated ion channels, collectively enhancing osteogenic differentiation. The Hippo pathway is activated by increased cytoskeletal tension and regulates mechanosensitive transcription factors, such as YAP/transcriptional coactivator with PDZ-binding motif (TAZ), which in turn promotes the expression of osteogenesis-related genes, including bone morphogenetic protein 2 (BMP2), osteopontin (OPN), and osteocalcin (OCN). Similarly, stretch-activated ion channels respond to the mechanical strain of the substrate by modulating the intracellular calcium ion flux, which activates osteogenesis-associated signaling cascades. In the context of the Wnt/β-catenin signaling pathway, YAP and TAZ are key regulators of cell growth and tissue homeostasis. The inhibition of the β-catenin destruction complex and the subsequent accumulation of β-catenin in the nucleus can promote the expression of YAP/TAZ target genes, as both pathways are often coupled to regulate cell proliferation and survival.

Similarly, Sun *et al.* elucidated enhanced stem cell mechanotransduction in curved nanofiber networks, as opposed to conventional straight nanofiber networks 101. Curved fibers promote cell boundaries to cross several curved fibers, forming non-adhesive bridges, and actomyosin stress fibers generate strong intracellular tension, enhancing mechanotransduction. This mechanical signaling activates ion channels, such as Piezo1, on the cell membrane, inducing calcium ion influx, which influences transcriptional activity and promotes osteogenic differentiation. The curved fibers created stronger mechanical tension than that of the straight fibers and played a key role in osteogenesis. They observed the upregulation of osteogenic gene markers [Runx2, alkaline phosphatase (ALP), and OCN] and widely distributed cell bridges, which initiated mechanosensing and mechanotransduction signaling pathways.

#### Porous structures

The porous structure within scaffolds plays a critical role in supporting cellular functions by facilitating nutrient supply and promoting vascularization. The interconnected pores within the scaffold create pathways for the transport of essential nutrients, oxygen, and waste products, ensuring that the cells receive the necessary conditions for growth and survival. This is particularly important in TE applications where scaffolds are designed to mimic the natural ECM and promote tissue regeneration. Additionally, the physical properties of the porous structure, such as mechanical stiffness and pore size, provide cells with mechanical cues that can influence their behavior (Figure 3C) 103, 104. The physical stimuli exerted by the porous scaffold can trigger cellular responses, such as migration, proliferation, and differentiation, leading to improved tissue formation. These mechanical cues are vital for guiding stem cells toward the desired tissue phenotype, making porous scaffolds an effective tool in regenerative medicine.

Koo and Kim fabricated a collagen foam, which is highly porous and forms a collagen fiber network, efficiently inducing cell-cell interactions (Figure 3C) 103. When cells settle in this porous structure, they interact with one another, enhancing the communication between cells and influencing their behavior. Specifically, the notch signaling pathway, which facilitates intercellular communication, is activated and plays a crucial role in cell function and tissue formation. The highly porous structure allows cells to interact more closely, activating the Notch signaling pathway through the jagged canonical Notch ligand 1 (JAG1) and Notch 1 receptor (NOTCH1). As a result, Notch signaling is transmitted to the nucleus, where it activates the expression of target genes [hes family bHLH transcription factor 1 (*HES1*) and hes related with YRPW motif-like protein (*HEYL*)] related to cell differentiation and tissue formation. This interaction demonstrated that the highly porous structure promoted cell-to-cell communication, thereby facilitating osteogenesis more effectively. Moreover, the use of porous scaffolds has been extended to neural stem cells (NSCs). Kourgiantaki *et al.* effectively enhanced NSC delivery using porous, collagen-based scaffolds, improving neuronal differentiation and functional integration *in vivo* 105. This resulted in increased axonal elongation and decreased astrogliosis compared to those with the lesser or non-porous controls.

#### Hierarchical structures

Finally, hierarchical, multiscale topographies, as described by Yang *et al.*, have shown the potential to enhance osteogenic differentiation in human bone marrow-derived mesenchymal stem cells (hBMSCs) (Figure 3D) 106. The fabricated triple-scale scaffolds mimicking the collagen structure showed enhanced ALP, OPN, and YAP-TAZ expression markers as well as increased cell elongation in comparison to their double- and single-scale counterparts. However, although a combination of perpendicular- and parallel-positioned triple-scale fibers supported osteogenic differentiation, slight modifications in alignment, such as purely parallel levels, inhibited osteogenesis. This highlights the importance of fabricating corresponding angles for each level to fully exploit the effectiveness of the hierarchical scaffolds.

Koo and Kim (2024) developed a hierarchically porous collagen bioink with macro- and microscale pores 104. While the conventional use of porous structures has been correlated with substantial cell viability and a more robust cell mobility profile, the combination of macro- and micropores facilitates cell survival and proliferation, as well as cell stretching and spreading, respectively. hASCs in these hierarchically porous structures infused with BMP2 demonstrated both printability and bioactivity, eliminating the compromise between structural stability and cell activity, and subsequently inducing osteogenesis.

The results confirmed that inducing topographical cues through biomedical scaffold structures, whether aligned, curved, porous, or hierarchical, can substantially enhance the efficacy of stem cell therapy. Furthermore, the hybrid approaches described, such as using different pore sizes or combining fiber alignments, provide a more biomimetic strategy for improving TE outcomes. The use of fish-derived biomaterials to fabricate these scaffolds can further enhance their synergistic effects.

### Crosslinking strategies to enhance the structural integrity of fish-derived biomedical scaffolds

Fish-derived biomedical scaffolds are valued for their biocompatibility and bioactivity, positioning them as promising candidates for TE. However, compared to their mammalian counterparts, their limited structural integrity often restricts their performance. This limitation arises from their adaptation to aquatic environments, which can render materials such as collagen and dECM prone to denaturation at mammalian body temperatures 3, 107, 108. Specifically, Ahn *et al.* demonstrated that the denaturation temperature of collagen from tilapia and flatfish was 30 °C and 25 °C, respectively, while the denaturation temperature of porcine collagen was higher at 35 °C 109. The fiber tenacity of collagen from tilapia and flatfish was measured at 0.88 and 0.46 g/den, respectively, compared to 2.98 g/den for porcine collagen. Similarly, GelMA derived from cold-water fish skin exhibited larger pore diameters (198.1 ± 125.9 μm) and a lower compressive modulus (39.4 ± 1.7 kPa) compared to porcine skin (pore diameter = 141.1 ± 72.6 μm and compressive modulus = 93.6 ± 16.6 kPa) 110. Additionally, GelMA derived from fish skin had a faster degradation rate compared to porcine skin, with mass losses of 23% ± 0.8% and 28.1% ± 1.8%, respectively, after 21 days. These findings highlight the challenges related to the mechanical instability of fish-derived biomaterials, which may limit their application in tissue engineering.

To address these challenges, a range of crosslinking techniques has been developed to enhance scaffold stability. Photo-crosslinking employs light-activated bonding to increase rigidity, while chemical methods form covalent bonds between biomolecules, thereby improving mechanical strength. Enzymatic crosslinking, inspired by natural biological processes, provides a biocompatible and biologically relevant stabilization approach. Collectively, these methods enhance the durability and structural integrity of fish-based biomaterials, as detailed in the following section, while preserving the biocompatibility critical for TE applications.

#### Photocrosslinking

Photocrosslinking of biomaterials often relies on the methacrylation process, which introduces methacryloyl groups to free amines and hydroxyl groups to molecules that can undergo polymerization upon exposure to UV or blue light 111, 112. A critical requirement for this process is the use of free radical photoinitiators, such as irgacure 2959 (2-hydroxy-4′-(2-hydroxyethoxy)-2-methylpropiophenone) or lithium phenyl-2,4,6-trimethylbenzoylphosphinate. These photoinitiators generate free radicals when irradiated with UV or blue light, initiating the crosslinking of the methacryloyl species. Furthermore, the UV intensity and crosslinking conditions must be carefully selected to achieve appropriate cell viability because excessive irradiation can adversely affect cellular health.

Methacrylated gelatin (GelMA), derived from fish gelatin, facilitates photocrosslinking to produce hydrogels with tunable mechanical properties and controlled degradation rates for TE 113. Elkhury *et al.* further explored GelMA from cold-water fish skin as a potential biomaterial for skeletal muscle regeneration 113. The comparative study of the biological properties of fish- and porcine-derived GelMAs revealed that while both exhibited similar characteristics before crosslinking, fish-derived GelMA hydrogels demonstrated a higher mass swelling ratio and enhanced cell spreading after crosslinking. Moreover, GelMA synthesized from Greenland halibut skin in combination with methacrylated hyaluronic acid and chondroitin sulfate resulted in biomechanically stable scaffolds 114. Further investigation using ATDC-5 chondrocytes indicated their potential for cartilage tissue regeneration. Additionally, Cao *et al.*, have demonstrated an inverse opal film (IOF) patch was developed using a photo-crosslinking GelMA extracted from tilapia skin combined with chitosan and polyacrylic acid (PAA) to replicate colloidal crystal templates 115. The resulting IOF exhibited excellent biocompatibility, low immunogenicity, and antibacterial properties, while effectively promoting tissue growth and wound healing.

**Figure 4:**
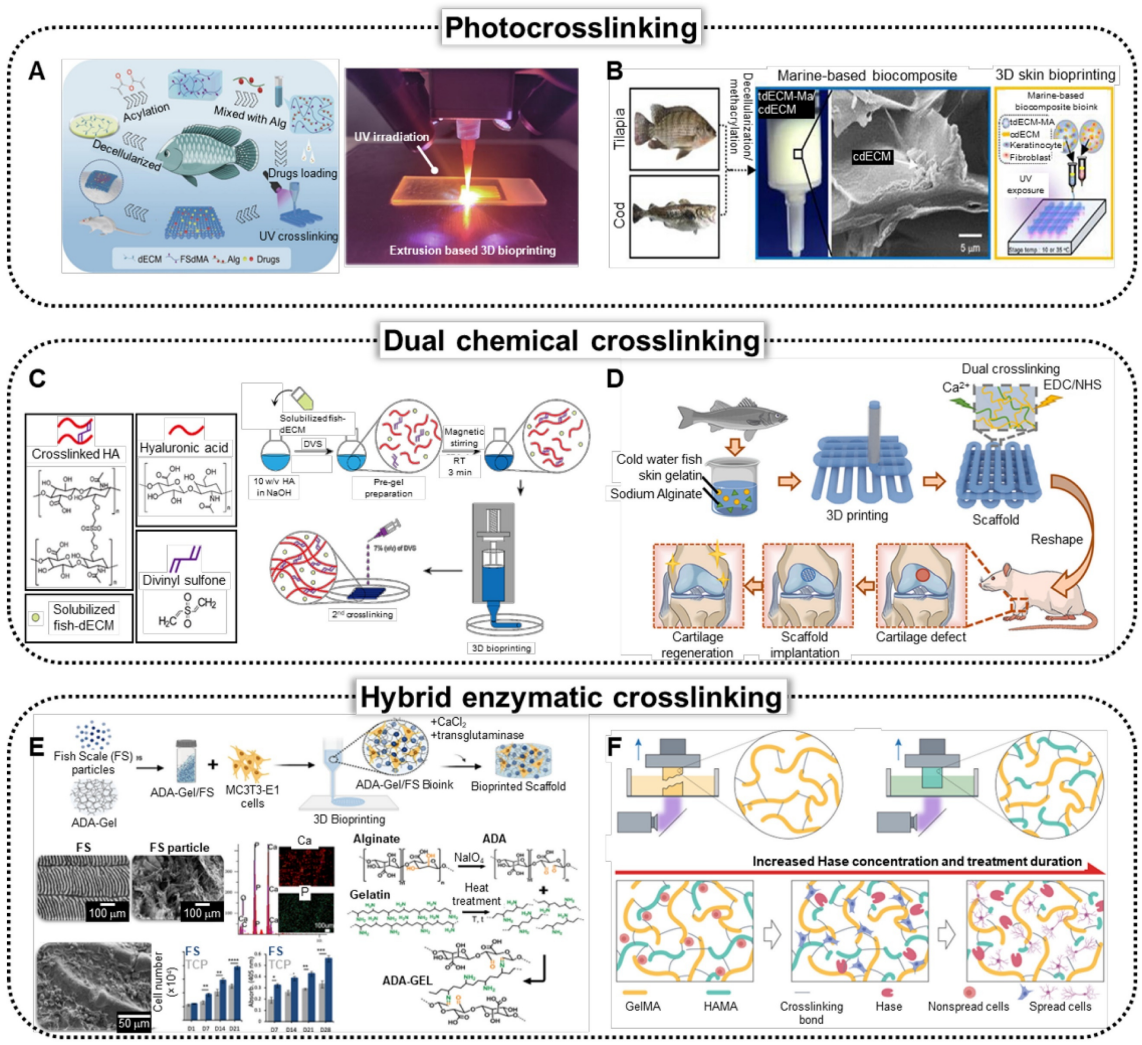
** Crosslinking strategies to enhance mechanical capabilities of fish-derived biomedical scaffolds.** (**A**) Schematics and optical image of the fabrication of the bio-printed fish-skin- decellularized extracellular matrix (dECM) methacrylate and application of skin regeneration (Adapted with permission from 116, copyright 2023). (**B**) Schematics of the bioprinting process of photocrosslinkable bioink composed of methacrylated tilapia dECM (tdECM-MA) and cod dECM (Adapted from 117, with permission). (**C**) Schematic of fish-derived dECM formulation and 3D hybrid bioink preparation (Adapted with permission from 26, 2023). Solubilized fish-dECM is pre-gelled using divinyl sulfone (DVS), followed by 3D printing to fabricate a structured model, with a secondary crosslinking step using DVS to stabilize the scaffold. (**D**) Schematics of fabrication of cartilage tissue therapeutics using cold-water fish skin gelatin scaffolds, demonstrating dual crosslinking of Ca^+^ and EDC/NHS crosslinking (Adapted with permission from 22, copyright 2023). (**E**) Schematics illustrating bioprinting of fish scale (FS) particles/alginate dialdehyde (ADA), which is crosslinked using CaCl_2_ and microbial transglutaminase (mTG) through ionic, covalent, and enzymatic methods. Characterization of FS through EDX analysis, and schematics of crosslinking mechanism (Adapted with permission from 123, copyright 2023).

The dECM can be methacrylated to produce photo-crosslinkable bioinks. A recent study by Lin *et al.* demonstrated the 3D bioprinting of methacrylated tilapia skin dECM, specifically for wound-healing applications (Figure 4A) 116. To ensure structural stability, researchers have methacrylated dECM (dECM-MA), resulting in excellent biocompatibility scaffolds that substantially accelerate the chronic wound healing process *in vivo*. dECM-MA extracted from tilapia skin demonstrates appropriate mechanical stability. However, tilapia skin generally contains low levels of omega-3 fatty acids, which are more abundant in saltwater fish. To address this deficiency, Lee *et al.* have proposed a composite bioink consisting of dECM-MA from tilapia skin and dECM powder from cod fish (Figure 4B) 117. *In vitro* assessments using HS-27 fibroblasts, HaCaT keratinocytes, and EA.hy926 endothelial cells showed improved keratinization and vessel formation compared with those of mammalian-based biomaterials.

#### Chemical crosslinking

The mechanical instability of fish-derived biomaterials can be substantially enhanced by dual-crosslinking strategies that incorporate covalent and ionic bonds. By combining these two crosslinking methods, the resulting scaffolds exhibit improved structural integrity and durability, which are crucial for applications in TE and regenerative medicine. Covalent cross-linking provides robust, long-lasting connections between polymer chains, whereas ionic bonding adds flexibility and dynamic properties, allowing biomaterials to better withstand mechanical stresses and deformation 118, 119. This approach not only enhances the overall mechanical performance of fish biomaterials but also contributes to their biocompatibility and functionality in various biomedical applications.

Figure 4C illustrates the incorporation of divinyl sulfone (DVS) into Korean amberjack skin-derived dECM and hyaluronic acid-based bioink, facilitating the formation of sulfonyl bis-ethyl crosslinks between biomolecules 26. This pre-gelation step enhances the printability of the bioink. Following the fabrication of the 3D scaffolds, the structures were submerged in DVS solutions, initiating a secondary crosslinking process to further stabilize the scaffold architecture. Through the *in vitro* biological evaluation of human dermal fibroblast (HDF) and HaCaT cells, the proposed methodology demonstrated an alternative solution to mammalian-based scaffolds. Figure 4D shows the dual crosslinking process of the bioink composed of cold-water fish skin gelatin and sodium alginate. The formulated bioink was 3D printed, followed by treatment with EDC/NHS solution and calcium chloride solution 22. EDC/NHS crosslinking formed stable covalent amide bonds between the carboxyl and amine groups, whereas calcium chloride-induced ionic crosslinking with sodium alginate created ionic bonds between the calcium ions and alginate. Rheological analysis demonstrated that the addition of sodium alginate substantially increased the viscosity of the bioink, leading to improved shear-thinning behavior, which facilitated the successful extrusion-type 3D printing of the composite bioink.

#### Hybrid enzymatic crosslinking

Enzymatic crosslinking enhances the mechanical stability of biomaterials by forming covalent bonds between polymer chains, creating a more rigid and cohesive network. This method improves structural integrity under external loading conditions and is commonly used in conjunction with other forms of crosslinking. Enzymes, such as transglutaminase 120, lysyl oxidase 121, and tyrosinase 122, catalyze the formation of stable covalent linkages between specific amino acid residues, such as lysine, glutamine, or tyrosine, within the protein or polymer matrix. These bonds lock the molecular chains into a more stable configuration, thereby preventing slippage and structural failure under mechanical stress. The improved stability reduces the risk of scaffold swelling or degradation in physiological environments, making the material more durable over time. Increased mechanical stability also allows the scaffold to provide mechanical cues to cells, supporting tissue regeneration while maintaining its shape and function during implantation.

Figure 4E shows the development of biomaterial inks that mimic the physicochemical properties of the ECM, which is crucial for advancing bioprinting technologies in TE 123. A composite biomaterial ink composed of FS and alginate dialdehyde (ADA) gelatin was created to enhance bioactivity and mechanical strength, and transglutaminase was used as an enzymatic crosslinking method along with calcium crosslinking of the alginate component to improve structural integrity.

Conventional crosslinking methods face limitations in balancing the physical performance of bioinks for high-fidelity tissue fabrication and creating suitable microenvironments for encapsulated cells. To overcome this issue, researchers introduced a molecular cleavage approach that utilizes hyaluronic acid methacrylate (HAMA) mixed with GelMA extracted from cold-water fish (Figure 4F) 124. The proposed method was followed by the selective enzymatic digestion of HAMA using the Hase enzyme to achieve tissue-matching mechanical properties while preserving structural complexity and fidelity.

## Stem cell therapy using fish-based biomedical scaffold

**Figure 5:**
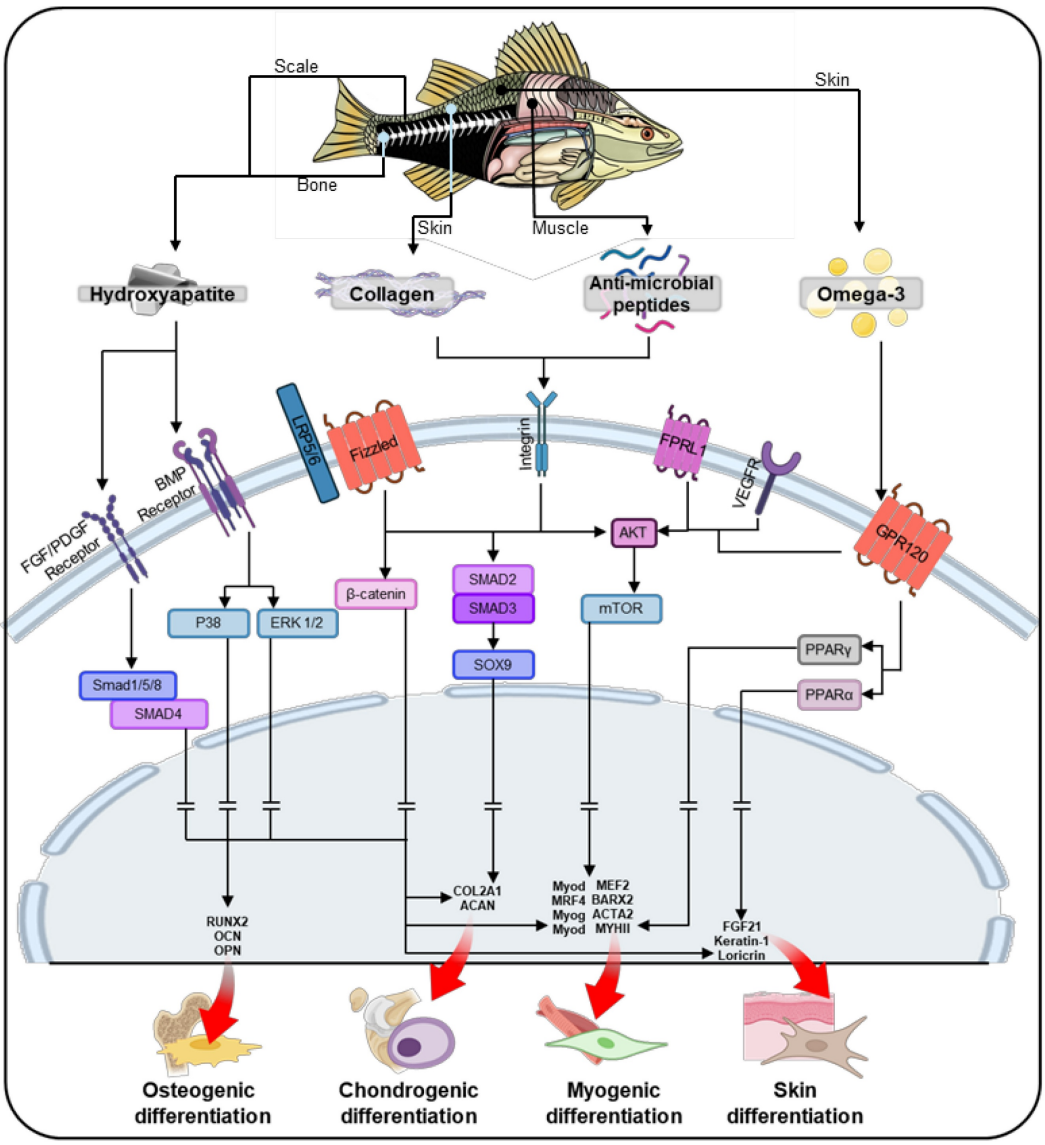
Schematic illustration of the stem cell lineage determination process through core constituents of fish-based biomaterials, including collagen, anti-microbial peptides, and omega-3 fatty acids, facilitating tissue-specific differentiation in osteogenesis, chondrogenesis, myogenesis, and skin differentiation.

**Figure 6:**
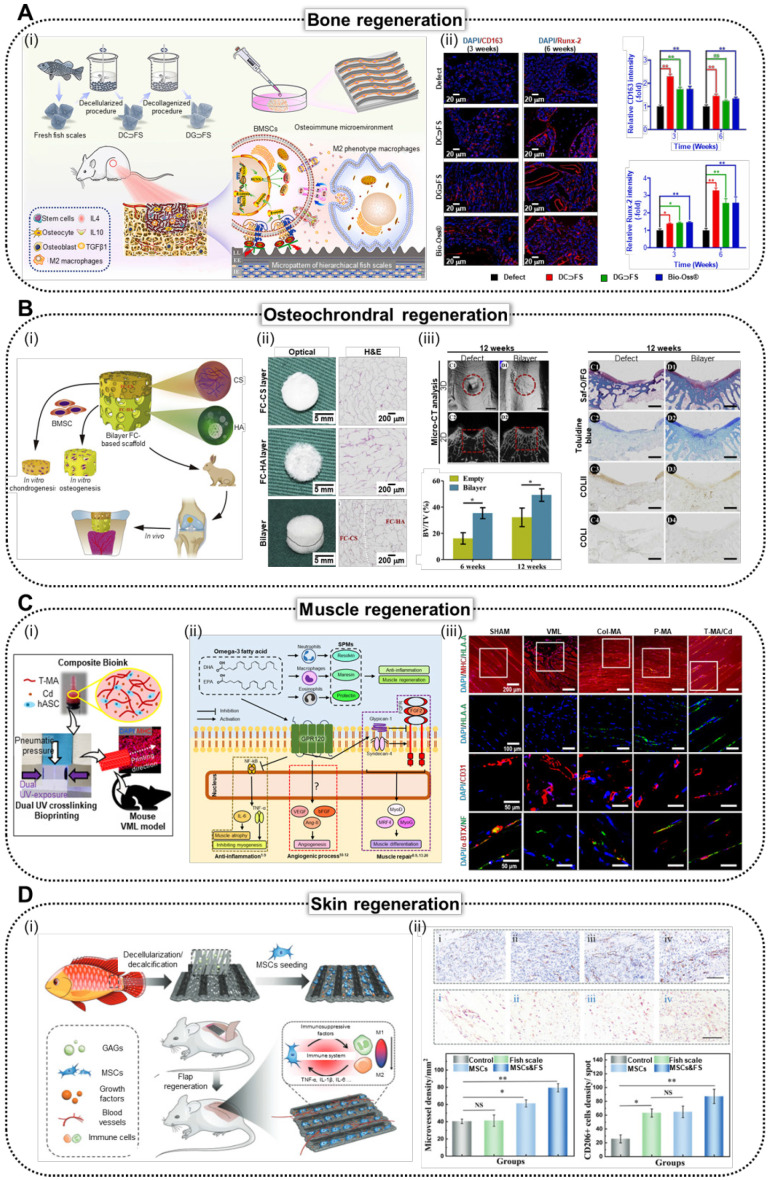
** Various stem cell applications using fish-based biomedical scaffolds.** (**A**) i. Schematic illustration of the natural micropatterned fish scales (FSs) designed to orchestrate cell behavior and the osteoimmune microenvironment, thereby enhancing regeneration of critical-sized bone defects. (ii) Immunofluorescence staining and (iii) quantitative analysis of the CD 163 (M2 macrophage polarization) and Runx2 (osteogenic markers) of CON (negative control), decellularized FS (DC⸧FS), decellularized/collagenized FS (DG⸧FS), and Bio-Oss^®^ (commercially available bone graft) (Adapted with permission from 34, copyright 2023). (**B**) (i) Schematics, and (ii) optical and H&E staining images of bilayer scaffolds composed of fish collagen (FC; hydroxyapatite integrated bottom layer, and chondroitin sulfate integrated top layer). (iii) *In vivo* results of implantation of bilayer FC-based scaffold into articular joint defect in rabbits demonstrating enhanced osteochondral regeneration. (Adapted with permission from 15, copyright 2020). (**C**) (i) Illustration demonstrating composite bioink composed of methacrylated tilapia decellularized extracellular matrix (dECM; T-MA) and cod dECM (Cd) and dual UV crosslinking systems. (ii) Biological responses of human adipose stem cells (hASCs) to omega-3 fatty acids and (iii) immunofluorescence staining of MHC, HLA, CD31, α-BTX, and NF of the muscles harvested from mouse that received SHAM (positive control), VML (negative control), Col-MA (methacrylated porcine skin-derived collagen), P-MA (methacrylated porcine skin-derived dECM), and T-MA/Cd (fish-based composite structure) (Adapted with permission from 21, copyright 2024). (**D**) (i) Scheme illustrating the fabrication of mesenchymal stem cell (MSC)-loaded FS scaffolds, highlighting their role in modulating immune responses by promoting M2 macrophage polarization and enhancing skin flap survival through reduced inflammation and improved tissue regeneration. (ii) Immunohistochemical staining of CD31 (vascularization marker) and CD 206 (M2 macrophage polarization marker) demonstrating enhanced vascularization and M2 macrophage polarization in rats that received control (negative control), FS, MSCs, and MSCs&FS (Adapted with permission from 12, copyright 2022).

Fish-based biomaterials activate intercellular pathways that play a crucial role in guiding stem cell differentiation. Key components such as collagen, hydroxyapatite, AMPs, and omega-3 fatty acids drive the differentiation of stem cells into osteogenic, chondrogenic, myogenic, and skin lineages (Figure 5). These biomaterials create a signaling environment that directs stem cells through tissue-specific pathways, promoting growth, proliferation, and differentiation. Additionally, scaffold design parameters significantly enhance the regenerative potential of fish-based biomaterials. Structural features such as alignment, curvature, porous, and hierarchical structures not only facilitate nutrient and oxygen transport but also guide stem cells through effective mechanotransduction, thereby enhancing their bioactivities. As shown in Table 2 and Figure 6, fish-derived biomaterials offer significant potential in stem cell therapy, steering differentiation toward targeted tissues. The following section explores their impact on tissue regeneration.

### Bone regeneration

Scaffold design plays a critical role in bone regeneration, with features such as porosity and curvature enhancing osteogenic activities in stem cells. Previous studies have demonstrated that scaffolds with porous 125 and curved structures 126, 127 effectively induce osteogenic differentiation, promoting bone-like tissue formation. Specifically, it has been reported that osteogenesis is most effectively promoted when the pore diameter ranges between 200 and 400 µm, as this enhances cell-cell interactions, cell migration, and proliferation. Additionally, compared to scaffolds with a smooth surface, those with a rough surface can stimulate focal adhesion of cells, thereby facilitating osteogenic differentiation 128. These characteristics are essential for fostering a favorable environment for bone regeneration.

In addition to scaffold design, the material components of fish-derived biomaterials significantly contribute to osteogenesis by activating key signaling pathways (Figure 5). Collagen plays a pivotal role in osteogenesis by activating the Wnt/β-catenin pathway, which promotes osteoblast differentiation and mineralization 129. Collagen binds to integrins on the cell membrane, triggering mechanotransduction that activates Wnt/β-catenin signaling 130. This process leads to the accumulation of β-catenin in the cytoplasm, which then translocates to the nucleus 131. This pathway is crucial for bone formation, as β-catenin regulates the expression of genes involved in osteogenic differentiation 132. Additionally, hydroxyapatite, especially in its nanoscale form, can activate the binding of bone morphogenetic protein (BMP) to BMP receptors 133-135. This activation leads to the phosphorylation of SMAD1/5/8, which then forms a complex with SMAD4. This SMAD complex subsequently upregulates the expression of osteogenic genes in the nucleus. AMPs further support osteogenesis by activating the Akt and mTOR pathways, enhancing osteoblast proliferation, survival, and mineralization 136. Omega-3 fatty acids reduce proinflammatory signaling and also activate the Akt/mTOR pathway, which promotes osteoblast differentiation and mineralization 137. Together, these components create a synergistic signaling environment that supports bone formation and regeneration.

Figure 6A shows the effectiveness of bone TE using decellularized/decollagenized *Lateolabrax japonicus* scales 34. The research team utilized anisotropic ridged micropatterns of FSs to modulate cellular orientation, induce macrophage polarization, and enhance osteogenic differentiation of hBMSCs through activation of the Wnt/β-catenin pathway. Through *in vivo* assessment of a rat femoral defect model, the research group demonstrated the capacity of FSs to induce M2 macrophage polarization, regulate the osteoimmune microenvironment, promote osteogenesis-related protein expression, and support bone matrix deposition and osteogenesis. This study highlighted FSs as promising bone fillers for accelerating bone regeneration. Additionally, Yu *et al.* investigated a novel 3D-printed fish gelatin methacrylate (FG) scaffold enhanced with strontium-doped calcium silicate powder (FGSr), which showed promising results for stem cell activation in bone TE 138. FGSr scaffolds not only exhibited 2.5-fold higher tensile strength but also enhanced the osteogenic differentiation and alkaline phosphatase activity of Wharton jelly derived mesenchymal stem cells (WJ-MSCs), positioning them as strong candidates for regenerative medicine applications. The research group further elaborated on the proposed method via the integration of BMP2, designed for enhanced drug delivery 139. The obtained structure demonstrated a stable localized release of BMP2, which substantially promoted cellular proliferation and increased alkaline phosphatase activity, indicating its potential for effective bone TE.

### Cartilage regeneration

The design of scaffolds plays a crucial role in enhancing cartilage regeneration, with hierarchical 140 and porous structures 141 proving particularly effective. These structural features have been shown to create biomimetic environments that accelerate cartilage formation by providing physical cues that promote stem cell differentiation and support tissue regeneration. According to previous documents, in articular chondrocytes, a pore size of 150-250 µm is considered suitable, whereas mesenchymal stem cells (MSCs) exhibit enhanced chondrogenic differentiation at pore sizes larger than 300 µm 142. Integrating such scaffold designs with fish-derived biomaterials further enhances the regenerative potential of cartilage tissue.

Fish-derived biomaterials promote chondrogenesis in stem cells through key signaling pathways (Figure 5). Through SMAD2/3 activation, collagen modulates chondrogenesis by upregulating the transcription factor SOX9, which enhances the expression of chondrogenic factors such as collagen type II alpha 1 chain (COL2A1) and aggrecan (ACAN), essential for cartilage formation 143. Additionally, AMPs regulate the Akt/mTOR pathway by binding to cell surface receptors, which activates Akt and downstream mTOR signaling, promoting protein synthesis and cell survival 144. This activation enhances chondrogenic differentiation by stimulating the production of cartilage-specific ECM components, such as collagen type II and aggrecan, essential for cartilage formation and maintenance 145. Omega-3 fatty acids help maintain ECM integrity, which is critical for cartilage formation, by interacting with the same Akt/mTOR pathways 144. This coordinated signaling environment supports the development of cartilage and the repair of joint tissues.

Diogo *et al.* investigated the potential of blue shark (*Prionace glauca*) skin collagen to induce chondrogenic differentiation of hASC using a cryogelation method to produce highly interconnected 3D constructs of collagen and hyaluronic acid 146. *In vitro* results demonstrated that hASC adhered well to these constructs, with early upregulation of chondrogenic markers, such as COL2A1 and SOX9, while the inclusion of hyaluronic acid enhanced the later expression of ACAN. These findings suggest that blue shark collagen can support initial differentiation; however, exogenous stimulation may be necessary to maintain the chondrogenic phenotype, making it a valuable biopolymer for cartilage applications.

Owing to advances in chondral regeneration, fish-based biomaterials have emerged as effective candidates for addressing osteochondral defects in the tissue regeneration process. As shown in Figure 6B, the bilayer FC-based composite scaffold consisted of an upper layer enriched with chondroitin sulfate to promote cartilage regeneration and a lower layer containing hydroxyapatite to facilitate bone formation 15. The scaffold architecture was designed to support the distinct microenvironments required for osteochondral tissue healing. Histological analysis revealed well-defined regions. The upper layer displayed organized cartilage-like tissue with an abundant ECM and cellular arrangements characteristic of chondrogenesis, whereas the lower layer exhibited robust bone formation, as evidenced by mineralized matrix deposition and the presence of osteocytes. These findings underscore the effectiveness of the scaffold in simultaneously promoting cartilage and bone regeneration, making it a promising candidate for osteochondral defect repair.

### Muscle regeneration

Alignment topographical cues are essential for guiding cellular orientation, a critical factor in promoting muscle regeneration. Scaffolds with aligned structures provide mechanical signals that direct stem cell alignment along the fibers, facilitating myogenic differentiation and enhancing muscle tissue formation 90, 147. Scaffold stiffness is also an important factor to consider in scaffold design. For skeletal muscle regeneration, a stiffness of approximately 12 kPa has been reported to be the most suitable 148.

Figure 5 also illustrates the key signaling pathways involved in myogenesis and the role of fish-derived biomaterials in activating these pathways. In myogenesis, collagen activates the SMAD pathway through TGF-β signaling, promoting myoblast fusion and differentiation, which are essential for muscle formation 149. The binding of collagen through integrins on the cell surface activates the TGF-β receptor and subsequent TGF-β/SMAD signaling 150. This pathway promotes myogenic differentiation by enhancing the expression of key myogenic transcription factors which direct stem cells toward muscle lineage commitment. AMPs further facilitate myogenesis by supporting protein synthesis and myoblast fusion through Akt/mTOR activation, which is crucial for muscle fiber formation 151. This activation also promotes myoblast fusion, a crucial step for forming multinucleated muscle fibers through inducing the fusion of myoblasts into more mature muscle fibers, a process that is essential for muscle regeneration 152. Omega-3 fatty acids enhance these processes by stimulating the Akt/mTOR pathway and peroxisome proliferator-activated receptor gamma (PPARγ) signaling, promoting protein synthesis and myoblast differentiation 153. Together, these molecules collaborate to guide stem cells toward muscle regeneration.

Recently, the effects of the aforementioned properties of tilapia and cod skin on skeletal muscles were evaluated (Figure 6C) 21. The biological effects of fish-based scaffolds (skin of tilapia and cod) were compared with those of mammal-based collagen and dECM using hASCs. Inflammatory markers were substantially downregulated in cells cultured in fish-based structures, whereas myogenic-related gene expression was upregulated. The implantation of volumetric muscle defects resulted in improved muscle regeneration and innervation in mice that received a fish-based scaffold.

### Skin regeneration

The incorporation of curved morphologies, resembling the natural rete ridge structure of the skin, is beneficial for effective skin regeneration. Scaffolds designed with such topographies not only mimic the skin's native architecture but also enhance the elasticity of the material, which is crucial for supporting tissue regeneration 154. The rete ridge structure of the basement membrane and the anisotropic structure can enhance the skin's elasticity 155. This increased elasticity enables the scaffold to better withstand the dynamic *in vivo* stresses during the regeneration period.

Figure 5 highlights the signaling pathways involved in skin differentiation in stem cells, showing how fish-derived biomaterials activate these processes. Collagen also contributes to skin differentiation by enhancing keratinocyte migration, proliferation, and differentiation through the Akt/mTOR pathway 156. Omega-3 fatty acids play a complementary role by supporting keratinocyte proliferation and epidermal barrier formation through the same signaling.

As shown in Figure 6D, Lin *et al.* demonstrated this effect by fabricating a biohybrid scaffold combining anisotropic FSs and human mesenchymal stem cells (hMSCs), preserving essential components, such as collagen and glycosaminoglycans 12. *In vivo* studies revealed that hMSC-loaded FS scaffolds effectively convert activated inflammatory macrophages into their anti-inflammatory forms, thereby reducing inflammation around the flap. These findings highlighted the potential of the scaffold to improve skin flap survival and functional recovery by modulating inflammation.

### Other tissue regeneration

Fish-derived biomaterials have promising applications in TE, particularly in supporting stem cell therapy across a range of tissues. Liu *et al.* recently suggested the activation of tendon-derived stem cells using tilapia skin dECM 157. The research group has demonstrated the upregulation of key proteins associated with tendon regeneration, including collagen I (Col-I), scleraxis (SCX), and tenomodulin (TNMD), as well as proteins associated with integrin α2/β1 and TGF-β/SMAD signaling pathways, suggesting the tilapia skin dECM can actively stimulate stem cells in tendon repair through integrin and TGF-β signaling pathways. Additionally, the fabricated scaffold mimicked the alignment structure of tendon tissue and exhibited a maximum tensile stress of approximately 4.12 MPa. This mechanical strength and structure are suitable for tendon tissue regeneration, which requires high mechanical properties 157. A recent study demonstrated an innovative approach for autoimmune disease treatment using adhesive microcarriers made from fish skin ECM loaded with engineered hMSCs 158. Using a microfluidic device, fish ECM microcarriers with a high surface area, porosity, and precise size control were created, enhancing hMSC attachment. Engineered hMSCs express PD-L1, which activates the PD-1/PD-L1 pathway and suppresses immune cell activity and inflammation, making this combination a promising method for autoimmune disease therapy.

## Current challenges and future directions of fish-based biomaterials

Fish-derived scaffolds show significant promise in tissue engineering due to their biocompatibility, high collagen content, and sustainable sourcing. Compared to mammalian collagen, fish collagen offers advantages such as ease of extraction, lower disease transmission risks, minimal environmental impact, and fewer ethical concerns 159. Future research should focus on enhancing the mechanical properties of these scaffolds through crosslinking and blending synthetic polymers, as well as developing standardized extraction methods for clinical scalability. Emerging techniques like 3D bioprinting and nanotechnology could enable more complex scaffold designs, while integrating bioactive components such as growth factors may further enhance their regenerative potential. Long-term *in vivo* studies are essential to evaluate the safety, degradation, and efficacy of fish-derived scaffolds, paving the way for their broader clinical use.

### Crosslinking strategies and bioactivities

The application of fish-derived biomaterials in TE presents several interrelated challenges, each of which highlights crucial future research directions to enhance their effectiveness and reliability. A major concern is the crosslinking strategy required to stabilize fish-derived scaffolds. Figure 4 demonstrates that dual crosslinking often involves additional components, such as alginate, which, while structurally beneficial, may reduce the overall bioactivity of the resulting construct. This reduction in bioactivity poses a critical limitation, as the inclusion of fewer bioactive elements can hinder effective interactions between the scaffold and host cells, impacting key regenerative processes, such as cell adhesion, proliferation, and differentiation. Therefore, the development of novel cross-linking methods that avoid compromising bioactivity is an urgent area for further research. Such advancements would enable the creation of biomaterials with enhanced biological performance and foster greater regenerative capacity without sacrificing structural integrity.

### Key barriers to clinical translation of fish-derived biomaterials

Fish-derived biomaterials show great promise in tissue engineering and regenerative medicine, but their clinical translation faces challenges, particularly regarding the potential immune response due to their inherently xenogeneic nature 160. Immune rejection, including inflammation, encapsulation, or degradation of the biomaterial, can compromise therapeutic outcomes. Despite the favorable properties of fish-derived biomaterials, such as natural bioactivity and optimal degradation rates, immune incompatibility remains a primary obstacle to their clinical use. To overcome this, extensive preclinical and clinical studies are necessary to assess immune risks and develop pre-treatment protocols or modifications that enhance immune compatibility. By identifying and addressing these immune responses, researchers can improve the safety and efficacy of fish-derived biomaterials in clinical applications. A promising future direction is to utilize the inherent anti-inflammatory and antimicrobial properties of fish-based biomaterials. These characteristics could potentially be harnessed to modulate the host's immune response. Such advancements would enhance the clinical applicability of fish-derived biomaterials and expand upon therapeutic potential.

Another major challenge is the variability in the bioactive composition of fish-derived biomaterials, which is influenced by species, habitat, diet, and environmental conditions 161. Such variability affects essential properties, including mechanical strength, degradation rates, and bioactivity, ultimately impacting reproducibility and reliability 162. Standardizing extraction and manufacturing protocols is critical to ensure consistent quality and predictable therapeutic outcomes 163. In this regard, the development of lab-grown fish in controlled environments presents a promising solution, as it allows for the standardization of fish-derived biomaterials by controlling the composition of the diet and environment. This approach can reduce variability and enhance reproducibility. Moreover, scalability remains a significant hurdle for clinical applications. Research into cost-effective, large-scale production methods, such as bioreactors and tissue engineering techniques, is needed to overcome this challenge and facilitate the widespread use of fish-derived biomaterials.

Ethical concerns also arise in the development of fish-derived biomaterials, particularly regarding animal welfare. The methods used to harvest fish for biomaterial production can raise significant concerns about the treatment of these animals, especially if they are subjected to stress, injury, or poor living conditions 164. While fish-derived biomaterials tend to present fewer ethical dilemmas compared to mammalian alternatives such as those related to disease transmission, the humane treatment of fish is crucial. Adopting responsible sourcing practices, adhering to animal welfare guidelines, and minimizing harm during the research and production processes can help mitigate these ethical challenges while promoting sustainability and respect for the animals involved.

The translational barriers to clinical applications are substantial, with regulatory hurdles being a primary concern. Demonstrating the safety, biocompatibility, and efficacy of fish-derived biomaterials to meet the rigorous standards of regulatory agencies like the FDA (U.S. Food and Drug Administration) and EMA (European Medicines Agency) is essential. Additionally, addressing high production costs and market acceptance will be critical for commercialization. Collaborative efforts among researchers, industry, and regulatory bodies are needed to overcome these challenges and unlock the full potential of fish-derived biomaterials in clinical settings.

## Fish-derived scaffolds in cancer therapy

### Fish-based biomaterials as anti-cancer agent

Fish-derived biomaterials have gained attention as promising candidates for cancer therapy due to their potential to inhibit tumor growth 165-168. Compounds such as EPA, fish oil, hydrolysates, and collagen peptides have demonstrated significant cancer-inhibitory effects (Table 3). For example, collagen peptides derived from tilapia exhibited potent anti-cancer activity against A549 lung cancer cells, inducing dose-dependent apoptosis via caspase activation, causing G2/M cell cycle arrest, and demonstrating substantial cytotoxicity 46. Likewise, EPA selectively inhibited the proliferation of A549 cells by generating prostaglandin (PG)E3, which downregulated Akt phosphorylation, suppressed tumor growth, and reduced tumor weight by 58.8 ± 7.4% *in vivo* 169.

However, despite these promising findings, the *in vivo* validation of the specific mechanisms underlying the anti-cancer effects of fish-derived biomaterials remains limited. The precise pathways through which these biomaterials exert their therapeutic effects, as well as their interactions with the tumor microenvironment, are not fully understood. Future research should focus on elucidating these mechanisms through advanced preclinical models, while also addressing challenges such as optimizing biomaterial delivery, ensuring biocompatibility, and verifying long-term efficacy and safety. With further development, fish-derived biomaterials hold significant potential to advance as novel, mechanism-driven therapeutic agents for cancer treatment.

### Fish-based scaffolds as postsurgical cancer therapy

In postsurgical cancer therapy, tumor resection often leaves significant tissue defects that need to be promptly repaired to restore normal function and structure. The removal of malignant tumors disrupts surrounding tissues, including skin, muscle, and bone, leading to impaired healing and functional deficits. To address this challenge, fish-based scaffolds have emerged as a promising solution for enhancing tissue regeneration after tumor excision. Derived from bioactive components like collagen, gelatin, and other ECM materials from fish tissues, these scaffolds provide a biocompatible platform that supports cellular attachment and growth. Recently, fish-derived gelatin-based scaffolds with encapsulated curcumin have effectively promoted tissue regeneration after tumor resection 165. *In vitro* and *in vivo* studies demonstrated that the scaffold not only showed potent cytotoxicity against gastric cancer cells but also significantly enhanced tissue repair and regeneration at the surgical site. Similarly, Li *et al.* demonstrated that fish GelMA scaffolds with a well-ordered porous structure promote tissue regeneration and prevent tumor recurrence in gastric cancer models, offering biocompatibility that supports normal cell proliferation 170. These features highlight the potential of fish-based scaffolds in improving postoperative tissue repair and reducing tumor regrowth.

**Figure 7:**
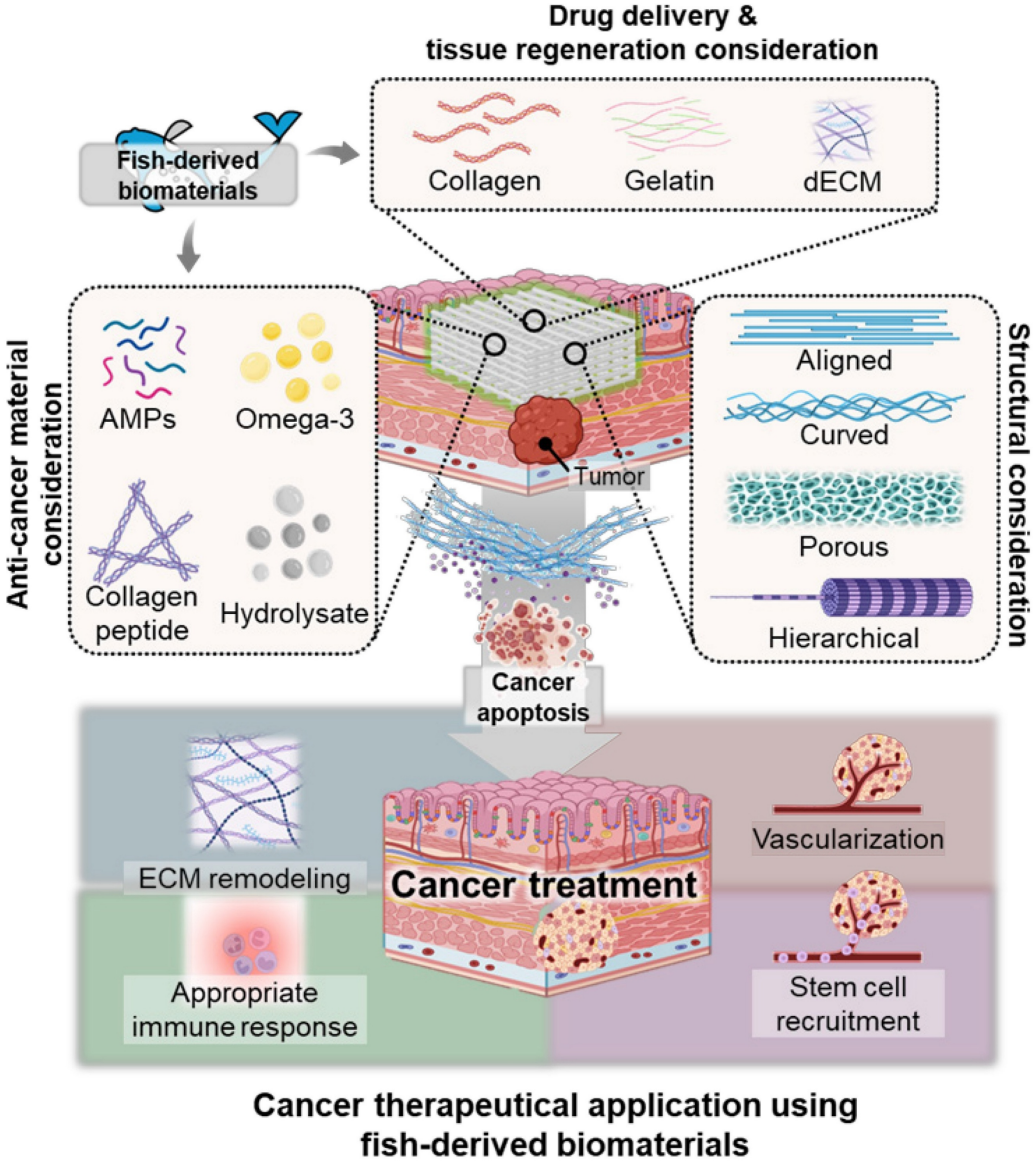
** Applications of Fish-Derived Biomaterials in Scaffold-Based Cancer Therapy.** Schematic illustrating the roles of fish-derived bioactive components and natural polymers in scaffold-based cancer therapy. The bioactive components include hydrolysates, AMPs (AMPs), and omega-3 fatty acids, each contributing distinct anti-cancer and immune-activating properties.

Currently, stem cells combined with bioengineered scaffolds are utilized in postsurgical cancer therapy to enhance tissue regeneration at tumor resection sites 171-173. However, the potential of incorporating stem cells into fish-based biomaterials to activate regenerative functions for more effective tissue repair at tumor resection sites remains unexplored. The next step for fish-derived biomaterials is to investigate their synergistic effects, focusing on both the bioactive properties of fish-based materials and the design of scaffolds, with the goal of significantly improving tissue regeneration and accelerating recovery in patients following cancer surgery. Future research should focus on integrating the anti-cancer properties of fish-derived biomaterials with advanced biofabrication technologies to enhance post-surgical cancer therapy. By coupling the anti-cancer bioactivities of fish-derived biomaterials with structural innovations in scaffold design, this integrated approach can effectively promote tissue regeneration, inhibit tumor recurrence, and accelerate recovery in patients following cancer surgery (Figure 7).

## Conclusions

Fish skin derived biomaterials show great promise for tissue regeneration owing to their ability to activate stem cells and support surrounding tissue repair. Abundant bioactive compounds, such as collagen, glycosaminoglycans, and omega-3 fatty acids provide structural integrity and biochemical signals that promote cellular attachment, differentiation, and anti-inflammatory responses. This capability makes fish skin particularly suitable for wound healing, muscle regeneration, and osteochondral defect repair. Beyond regenerative medicine, recent studies have suggested that fish skin biomaterials could serve as innovative platforms for cancer therapy and modeling. The biocompatible and structurally versatile scaffold of fish skin not only supports stem cell differentiation but also facilitates cancer research, providing a supportive environment for studying tumor-stroma interactions and testing therapeutic approaches. Collectively, the unique properties of fish skin make it a versatile biomaterial with broad applications in regenerative medicine and oncology.

## Figures and Tables

**Table 1:** Biofabrication strategies of fish-derived biomaterials

Fabrication Technique	Fish-derived Biomaterial	Fish Species	Target Tissue	Advantages	Drawbacks	Ref.
Soft lithography	Collagen (Type I)	Tilapia	Oral Mucosa	Similar rheological characteristics with mammals	Incomplete shape fidelity of scaffold-embedded microstructures	174
				Minimal shrinkage Excellent micropattern transfer	Crosslinker-dependent micropattern dimension	175
				Excellent micropattern transfer on scaffold surface	Collagen dehydration-induced shrinkage	176
Electrospinning	Gelatin	Salmon	Skeletal Muscle	Enhanced mechanical properties Superior cell differentiation	Differentiation of late myogenic markers still unexplored	177
		240 bloom type A (not specified)	General Tissues	Enhanced mechanical properties Superior cell differentiation	Poor retention of fibrous morphology	178
		Cold water fish skin (not specified)	Connective Tissues	Enhanced cellular activities	Crosslinker-dependent cell-adhesion and proliferation	179
				Enhanced cellular activities Low shear stress Enhance scaffold tunability	Crosslinker-dependent stability and mechanical properties	180
		Channel Catfish	General Tissues	Enhanced mechanical properties and structural stability	Hybrid material-dependent stability	181
	Fish Oil (Omega-3 Polyunsaturated Fatty Acids)	Not specified	General Tissues	Improved oxidative stability	Enzyme-dependent release profile	182
				Controllable fiber diameter by fish oil loading	High hydroperoxide content and secondary oxidation products	183
		Commercial Cod (liver)	General Tissues	Minor differences in fiber morphology	Low oxidative stability	184
		Commercial Cod (liver)	General Tissues	Non-cytotoxic Increased cell adhesion and proliferation Smaller nanofiber diameter	Weak mechanical properties	185
	Collagen (Type I)	Not specified	Thymopoeitic Moleculus	Non-cytotoxic Increased cell adhesion and proliferation	Weak mechanical properties	186
		Tilapia	Connective Tissues (fibroblasts)	Enhanced mechanical properties Antibacterial activity against Staphylococcus aureus	Decreased porosity with initial gelatin concentration	187
Freeze-Drying (Lyophilization)	Gelatin	Commercial Cod	Cartilage	Pore size control and high porosity Conducive cell adhesion and proliferation Non-cytotoxic	Changes in pore shape post-crosslinking	188
	Collagen (Type I)	Weever	Brain Tissue	Conducive cell adhesion and proliferation Non-cytotoxic	Low tensile modulus in non-hybrid scaffolds	189
		Tilapia	Skin Tissue	Pore size control and high porosity	Poor mechanical properties and structural instability	190
			Cartilage Tissue	Enhanced cellular activities High printability	Poor mechanical properties and structural instability	191
		Flatfish *(Paralichthys olivaceus)*	Skin Tissue	Pore size control and high porosity Non-cytotoxic	Rapid degradation without proper crosslinker	192
		Rohu and Catla	Bone Tissue	Pore size control and high porosity Conducive cell adhesion and proliferation Non-cytotoxic	Requirement for cell seeding procedure	193
Extrusion-based	Gelatin	Lizardfish *(Saurida spp.)*	Skin Tissue	Enhanced cellular activities	Poor mechanical properties and structural instability	139
				Enhanced cellular activities High printability	Poor mechanical properties and structural instability	194
		Not Specified	Bone Tissue	Enhanced cellular activities	Poor mechanical properties and structural instability	195
	Collagen (Type I)	Blue grenadier fish (*Macruronus novazealandii*)	Connective Tissues (fibroblasts)	High cell viability Lower shear stress Enhance scaffold tunability Readily purified	Low denaturation temperature	194
		Flatfish *(P. olivaceus)*	Bone Tissue	Enhanced cellular activities	Low structural stability	196
				Increased mineralization Substantial ALP activity Enhanced cellular activity	Low structural stability	197

**Table 2:** Strategies of stem cell therapeutical applications using fish-derived biomaterials

Target Tissue/Organ	Fish Derived Biomaterial	Fish Species	Biofabrication Method	Stem Cell	Advantages	Limitations	Ref
Bone	Gelatin	Non-specific	Extrusion Bioprinting	WJ-MSC	ALP upregulation	FGSr degraded faster compared to pure FG batches	138
	Gelatin	Non-specific	Extrusion Bioprinting	WJ-MSC	WJ-MSC differentiation and proliferation Sustained release of BMP-2	Aggregation on the FGSrB scaffolds by the rough surface	139
	Decellularized Fish Scale / GelMA from Fish-derived Gelatin	Gilt-head bream / Non-specific	Nanosheets	hMSCs	Higher mineralization by NIR light Fish-scale scaffold enhanced MSC proliferation and osteogenesis No immunological reaction detected	MSC proliferation on fish scales and GelMA lower than control disks	198
	Hydroxyapatite	Labeo rohita and Catla catla	Polymer Gel Casting	MSCs	MSC proliferation and adhesion with spread morphology	10-15% weight loss at 800-1,300 °C indicates HAp decomposition and OH- loss.	199
	Decalcified fish scales	Black carp (*Mylopharyngodon piceus*)	Freeze-Drying	BMSCs	CS-FS showed excellent mechanical properties Promoted differentiation of various cell types	Co-culture (BMSC/TSPC 1:1, 2:1) reduced TSPC tenogenic gene expression (DCN, COL1, and BGN)	200
Cartilage	Collagen	blue shark	Freeze-Drying	hASC	Hyaluronic acid incorporation boosts chondrogenesis support	Substantial cell-mediated contraction Enhanced chondrogenesis reduces scaffold stiffness	146
	Collagen	Eel	Extrusion bioprinting	hUMSCs	Collagen improves cell adhesion and proliferation	Reduced scaffold stability after printing​	201
Pancreas	Gelatin	Non-specific	Electrospinning	WJ-MSC	WJ-MSCs on Fish gelatin/PCL scaffold differentiated into insulin-producing cells Substantial insulin and C-peptide production in high glucose setups	Insulin and C-peptide production also substantial in low glucose setups Still unexplored Glut-2 function Need for in vivo studies	202
Muscle	Decellularized extracellular matrix	Tilapia and cod	Extrusion Bioprinting	ASC	Fish skin dECM has a lower risk of immune rejection Enhanced muscle differentiation and regeneration​	Low denaturation temperature Reduced stability	21
Periodontal Tissue (Gingiva)	Hydroxyapatite	Snapper and Salmon	Peptide Nanofibers	iPSCs	Salmon-scale HAp-hybrid scaffold showed higher mechanical strength	Species-specific morphological differences in fish-derived HAp	203
Skin	GelMa from Fish-derived Gelatin	Non-specific	Extrusion bioprinting	ASCs	Insignificant immune rejection or foreign body response​	Low cell viability (42.9 ± 9.1%) was observed on day 1	204
Thymus	Collagen	Non-specific	Electrospinning	HPSCs	Expression of thymopoietic genes and proteins	FC/PCL 0.4:9.6 scaffold showed slight increase in cell proliferation compared to PCL scaffolds	205

**Abbreviations** Wharton jelly-derived mesenchymal stem cell (WJ-MSC); alkaline phosphatase (ALP) ; fish gelatin methacrylate with strontium-doped calcium silicate powder (FGSr); bone morphogenetic protein 2 (BMP-2); BMP-2-loaded FGSr (FGSrB); human mesenchymal stem cells (hMSC); near-infrared (NIR); mesenchymal stem cell (MSC); hydroxyapatite (HAp); bone marrow mesenchymal stem cell (BMSC); calcium silicate-bioactivated fish scale (CS-FS); tendon stem/progenitor cells (TSPC); decorin (DCN); COL1A1 (COL1); biglycan (BGN); human adipose stem cell (hASC); human umbilical cord derived mesenchymal stem cell (hUMSC); glucose transporter 2 (Glut-2); induced pluripotent stem cell (iPSC); human pluripotent stem cell (HPSC); polycaprolactone (PCL); adispose stem cell (ASC); decellularized extracellular matrix (dECM); fish collagen (FC)

**Table 3:** Fish-based scaffold application in cancer therapy

Cancer therapy	Fish Derived Biomaterial	Fish species	Target cancer tissue	Evaluation	Key results	Limitations	Ref
Anti-tumor agent	EPA	-	Lung	*In vitro* (A549 and H1399 cells) *In vivo* (mouse xenograft tumor model)	Anti-proliferative activity of EPA in Cox-2 expressing cancer cell Generation of prostalandin E3 resulted in downregulation of Akt/PKB pathways	Anti-cancer effects of EPA dependent on COX-2 expression	169
	Fish oil	-	Lung	*In vitro* (A549 and H1399 cells)	Reduced cancer stem cell traits Reversed cisplatin resistance in A549 sphere cells Suggested a potential strategy for overcoming drug resistance in lung cancer	Unclear mechanisms for anti-cancer effects Insufficient *in vivo* validation	206
	Fermented fish bone using Monascus purpureus	*Chanos chanos*	Colon	*In vitro* (HCT-116 cells)	Anti-oxidant and anti-inflammatory properties Induced cell cycle arrest, Apoptosis and autophagy in human colorectal cancer cells	Unclear mechanisms for anti-cancer effects and insufficient *in vivo* validation	207
	Fish skin hydrolysate	*Oncorhynchus mykiss*	Colon	*In vitro* (HCT-116 cells)	Anti-oxidant effects of various bioactive peptides Highest anti-cancer effects using fractions of less than 3kDa	Unclear mechanisms for anti-cancer effects Insufficient safety about normal cell and in vivo validation	208
	Collagen peptides	Tilapia (*Oreochromis niloticus*)	Lung	*In vitro* (A549 and MRC5 cells)	More effective at inhibiting the growth of cancer cells than normal cells Induced G2/M arrest, necrosis, late apoptosis, Activated caspase-3, -8, and -9 of cancer cells in a dose-dependent manner	Insufficient *in vivo* validation Effects cell viability of normal cells depending on the dosage	46
	CPS fish-gut bacterium	-	Colon	*In vitro* (CaCo-2 and HCT-116 cells)	Activated Capases 3 and 9, suggesting a potential apoptosis in colorectal cancer cells.	Variability in anti-cancer effects depending on cell type Unclear mechanisms for anti-cancer effects Insufficient safety data for normal cell and *in vivo* validation	209
Drug delivery	Gelatin	-	Stomach	*In vivo* test using curcumin encapsulated by methacrylated fish gelatin	Continuous drug release process inhibits tumor growth and facilitates tissue regeneration	Insufficient further research to confirm the sustained therapeutic effect after implantation Lack of pharmacokinetics data in human	165
				*In vivo* test using 3D-printed fish GelMA scaffold loaded with berberine	High anti-cancer effect with high cell viability of normal cells Decrease of the tumor volume and inducing attachment and proliferation of normal cells for tissue regeneration Enhancing IL-17 and NOD-like receptor signal pathway	Unclear mechanisms for anti-cancer effects More verification needed using nude mice Lack of pharmacokinetics data	170

Abbreviations: eicosapentaenoic acid (EPA); cyclooxygenase-2 (COX-2); capsular polysaccharide (CPS); glioblastoma (GBM); Patient-derived xenograft (PDX)
